# Supplementary figures and images for: Mathematical model for the role of multiple pericentromeric repeats on heterochromatin assembly
Source: PLoS Comput Biol. 2024 Apr 10;20(4):e1012027. doi: 10.1371/journal.pcbi.1012027 (PMC11034663; doi:10.1371/journal.pcbi.1012027)

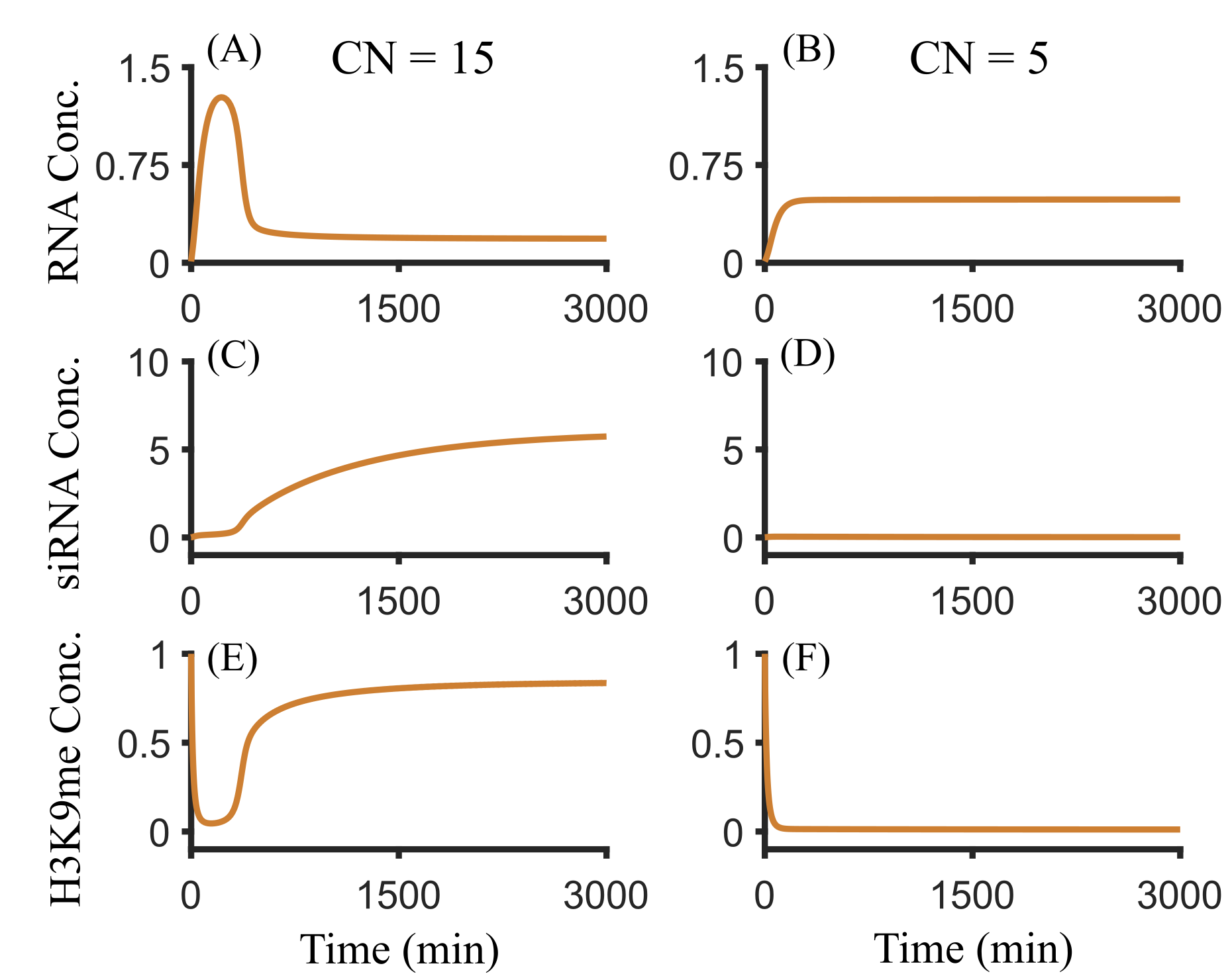

Supplement: S1 Fig — ODE solution of Eqs 1–3 for (A, C, E) CN = 15 and (B, D, F) CN = 5 for the same initial condition. (A, C, E) When CN = 15, the system is silenced, which is represented by high steady-state H3K9me concentration. (B, D, F) when CN = 5, the system is desilenced, which is represented by low steady-state H3K9me concentration. (TIFF) [file pcbi.1012027.s001.tiff]

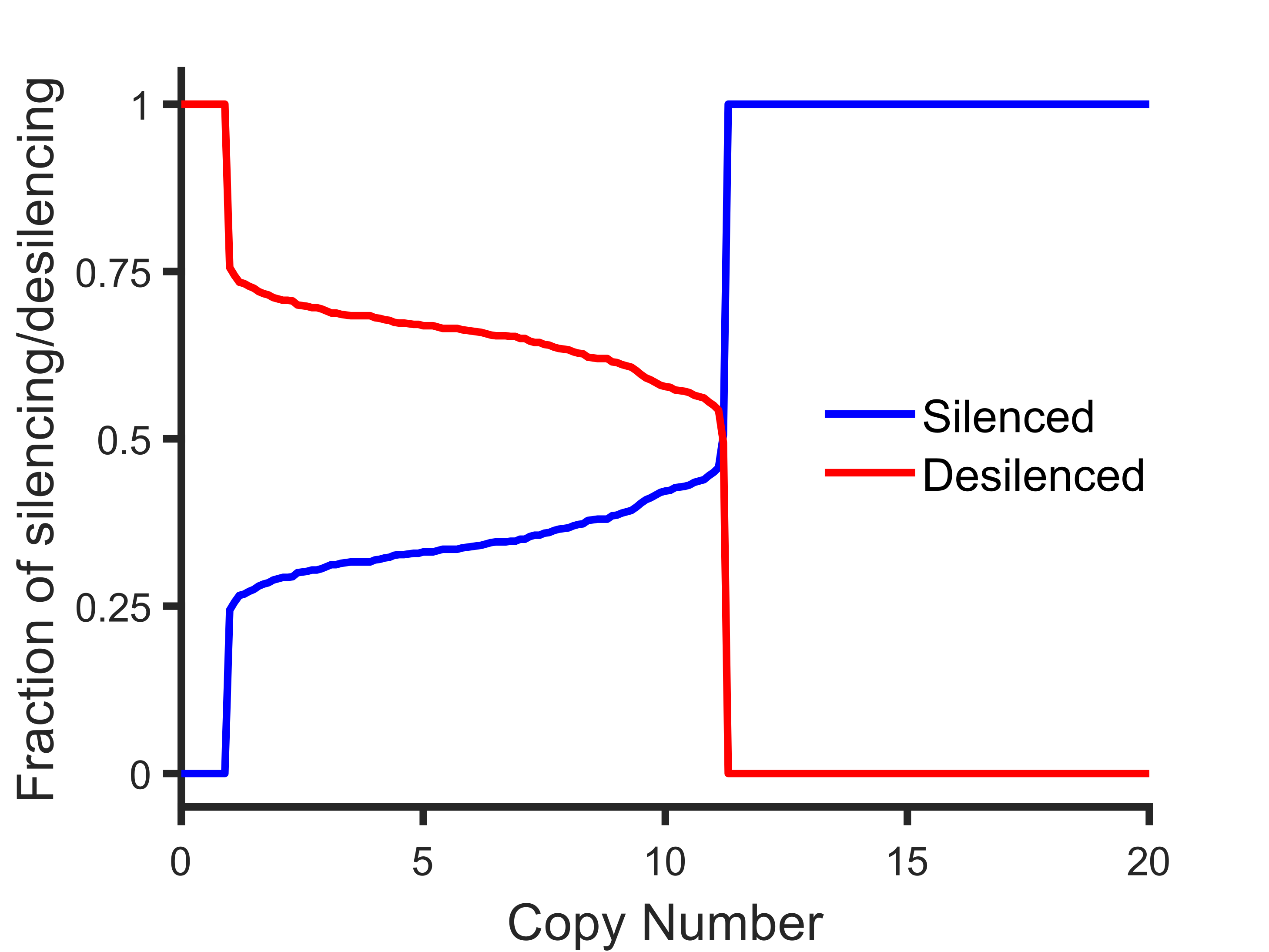

Supplement: S2 Fig — Bistable region is from CN = 1 to CN = 11. (TIFF) [file pcbi.1012027.s002.tiff]

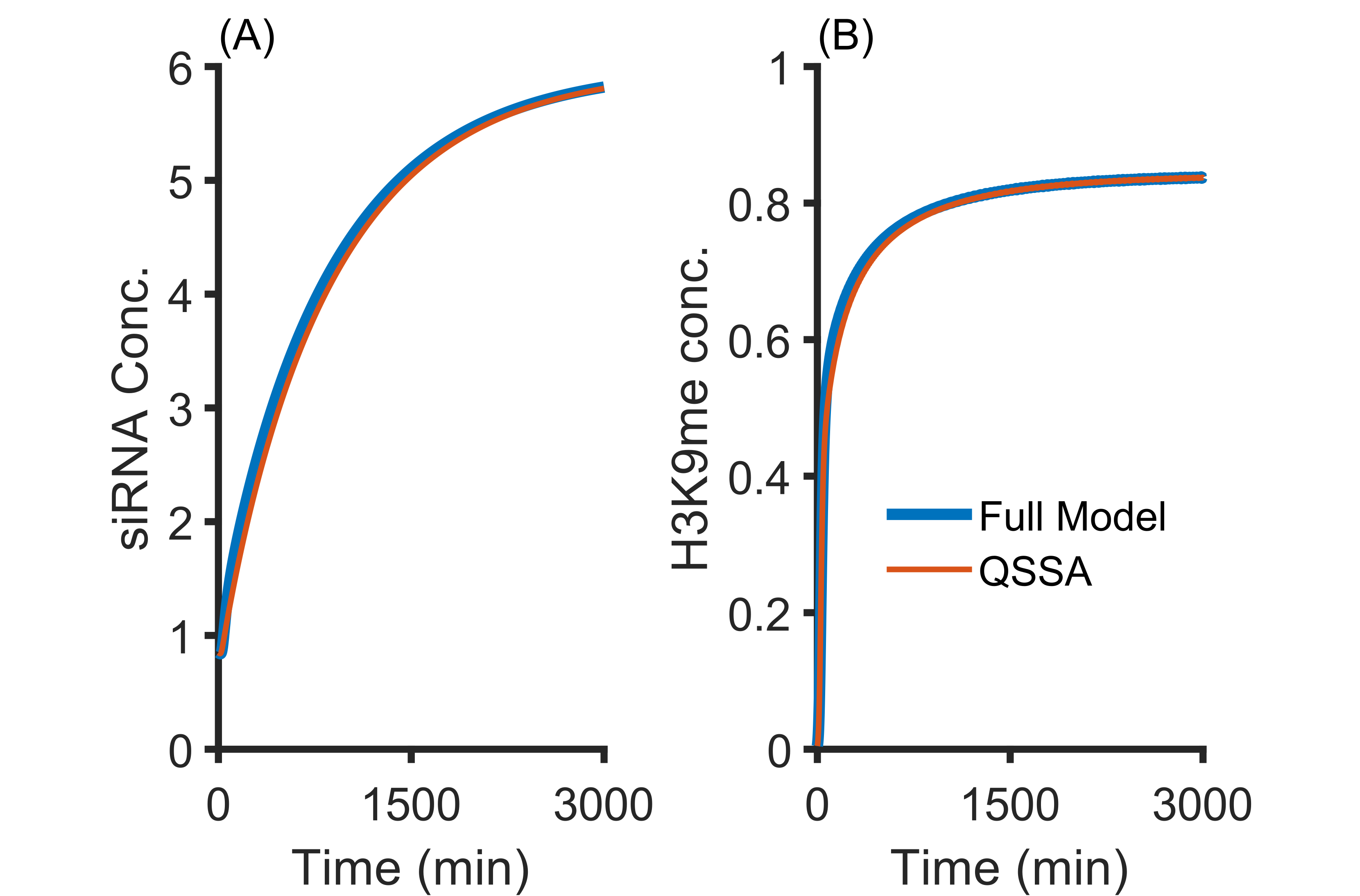

Supplement: S3 Fig — Comparison of time evolutions of (A) siRNA, and (B) H3K9me between full model and QSSA at CN = 15. Both siRNA (A) and (B) H3K9me concentrations evolve at similar rates between the full model and QSSA. (TIFF) [file pcbi.1012027.s003.tiff]

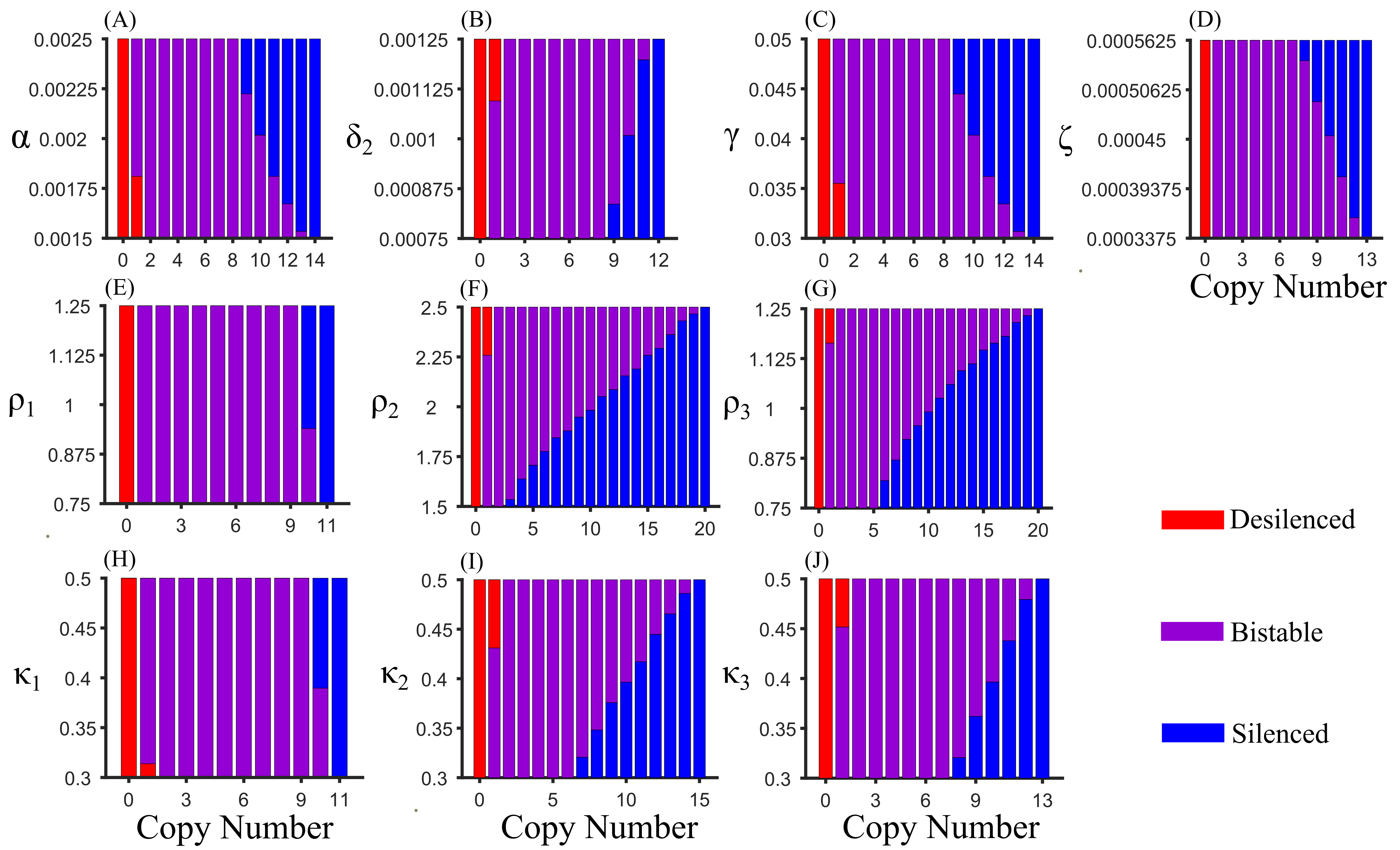

Supplement: S4 Fig — Other than one parameter, all other parameters are held at the default values. High values of (A) α, (C) γ, (D) ζ, (E) ρ1, and (H) k1 favor silencing, whereas high values of (B) δ2, (F) ρ2, (G) ρ3, (I) k2, and (J) k3 favor bistability or desilencing. α = Transcription Rate, δ2 = siRNA degradation rate, γ = siRNA biogenesis rate, ζ = Basal methylation rate, ρ1, ρ2, and ρ3 = Hill coefficient for transcription, siRNA biogenesis, methylation by siRNA respectively. k1, k2, and k3 = Half maximum methylation for transcription, siRNA biogenesis, and methylation by siRNA respectively. (TIFF) [file pcbi.1012027.s004.tiff]

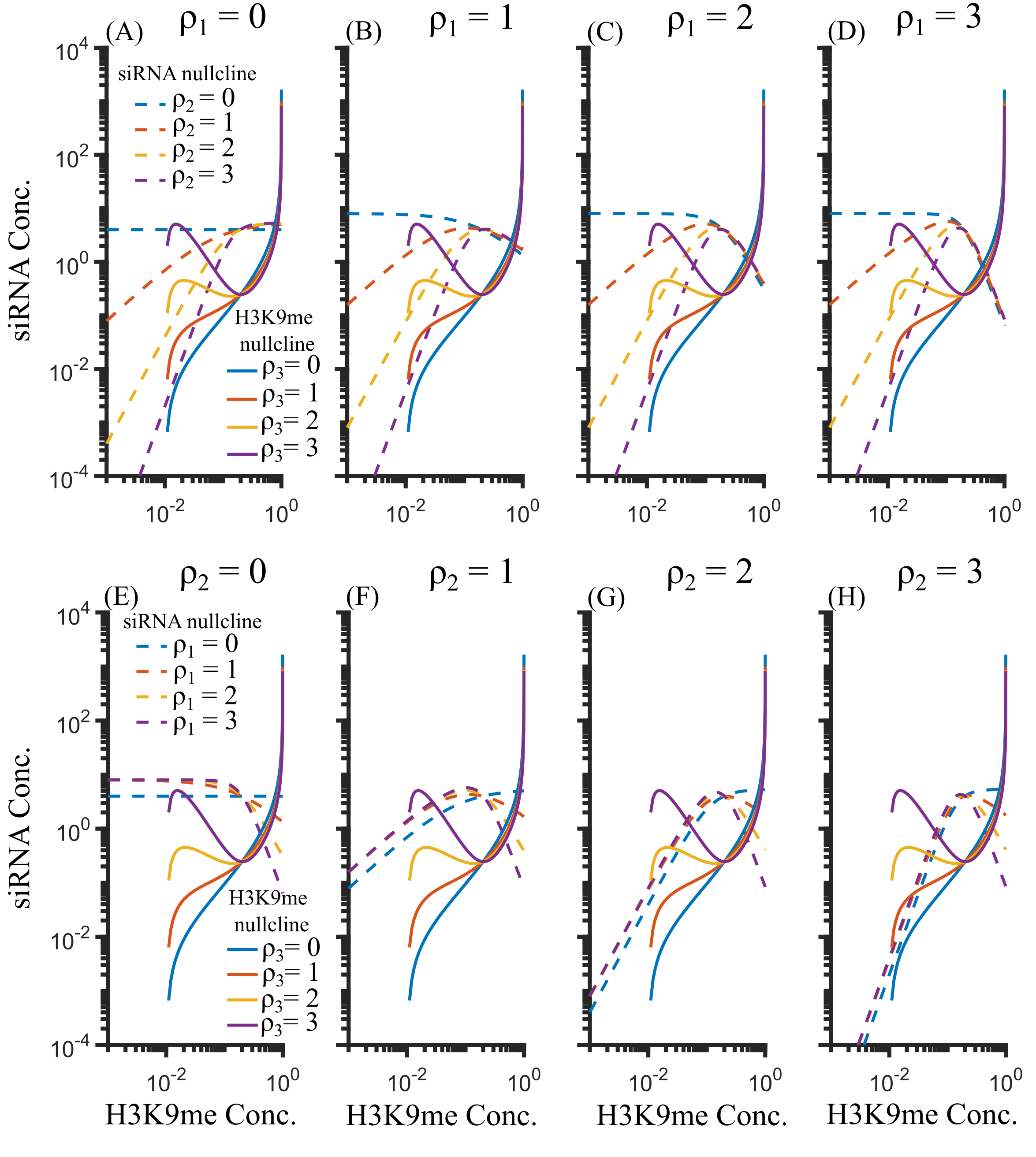

Supplement: S5 Fig — H3K9me nullcline and siRNA nullcline can intersect at three distinct points if ρ1≥1, ρ2≥2, and ρ3≥1 in the reference parameters. (TIFF) [file pcbi.1012027.s005.tiff]

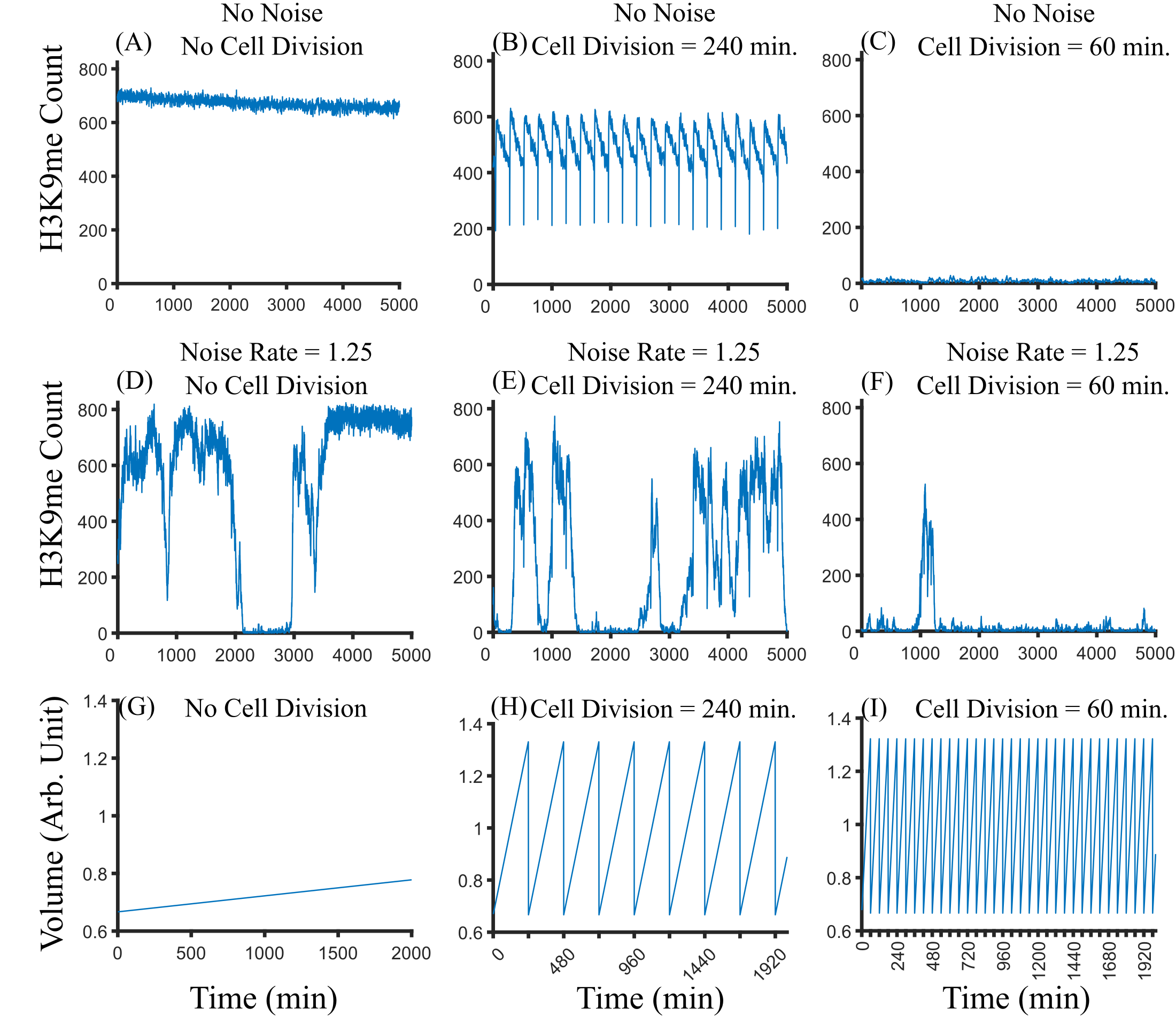

Supplement: S6 Fig — (A) Silenced state is favored for the system with no cell division and noise. (B-C) Cell division can lead to desilencing. (D-F) The H3K9me profile with noise. (G-I), the volume growth with respect to time, for different cell division of the system. (TIFF) [file pcbi.1012027.s006.tiff]

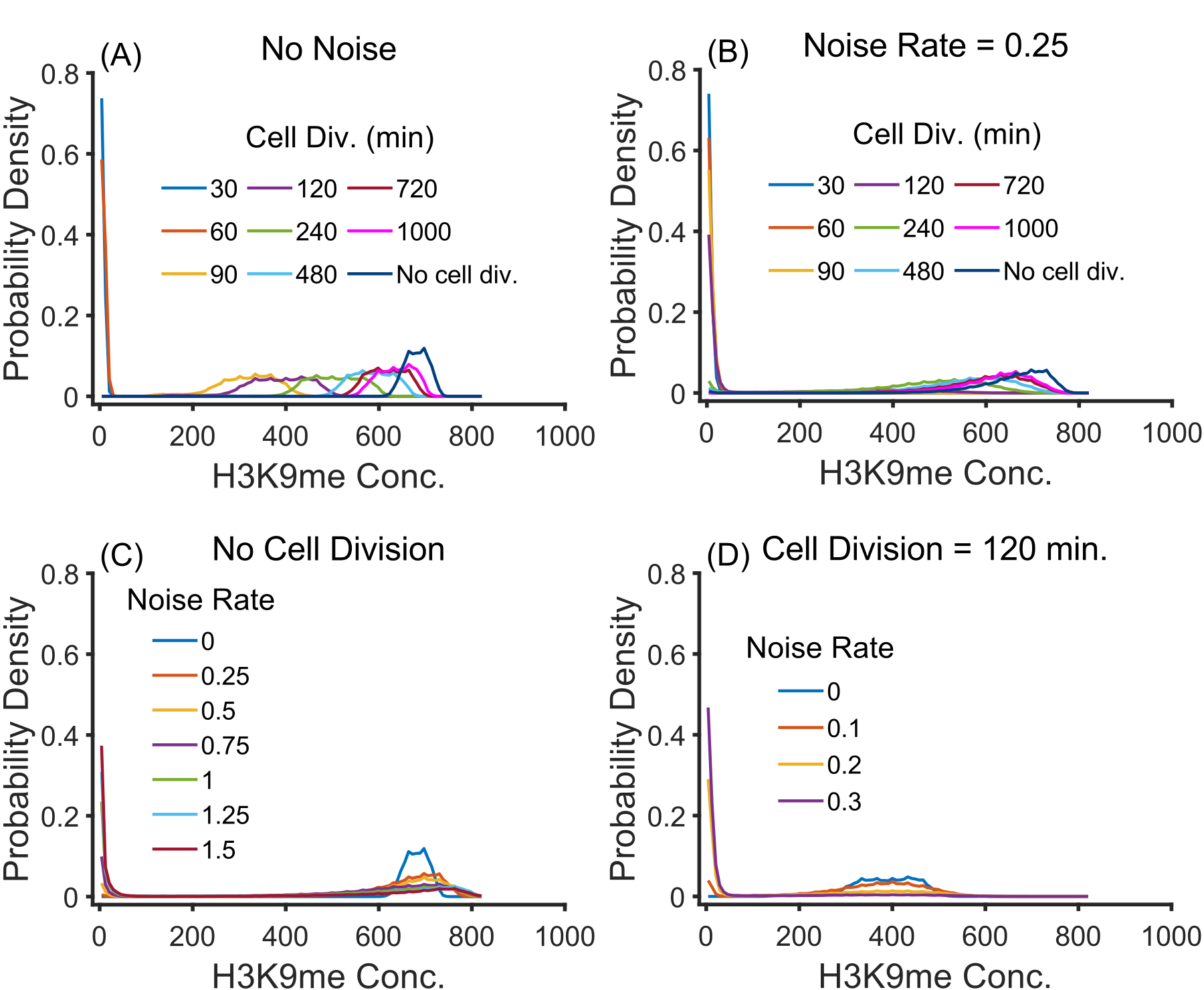

Supplement: S7 Fig — Faster cell division favors the desilenced state for a system (A) without noise and (B) with noise. Increase in noise brings the system down to desilenced state (C) without cell division and (D) with cell division. (TIFF) [file pcbi.1012027.s007.tiff]

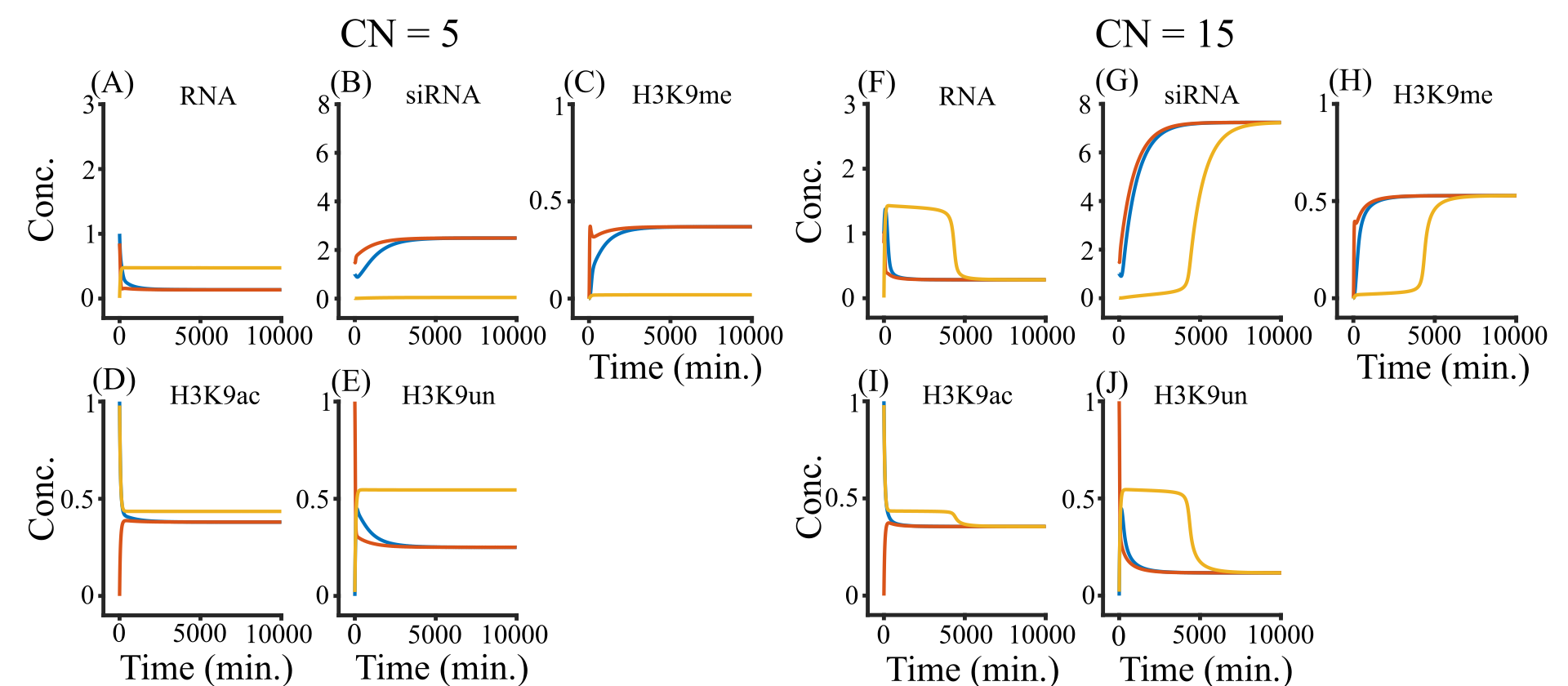

Supplement: S8 Fig — (A-E) ODE solutions when copy number is 5. (F-J) ODE solutions when copy number is 15. Colors indicate the use of different initial conditions. (PNG) [file pcbi.1012027.s008.png]

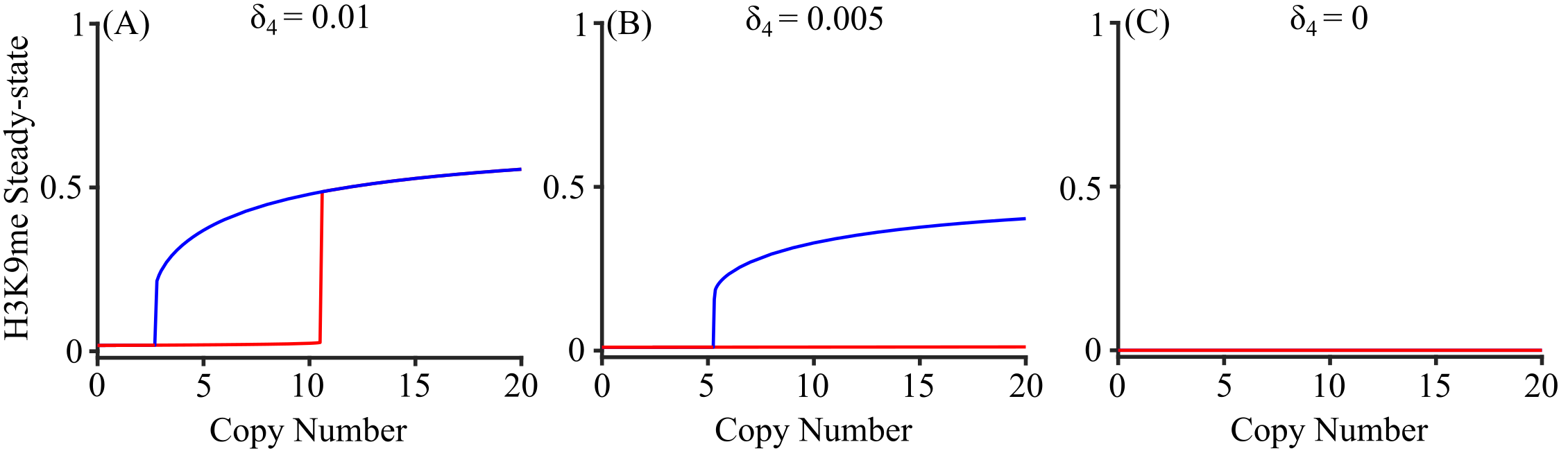

Supplement: S9 Fig — (PNG) [file pcbi.1012027.s009.png]

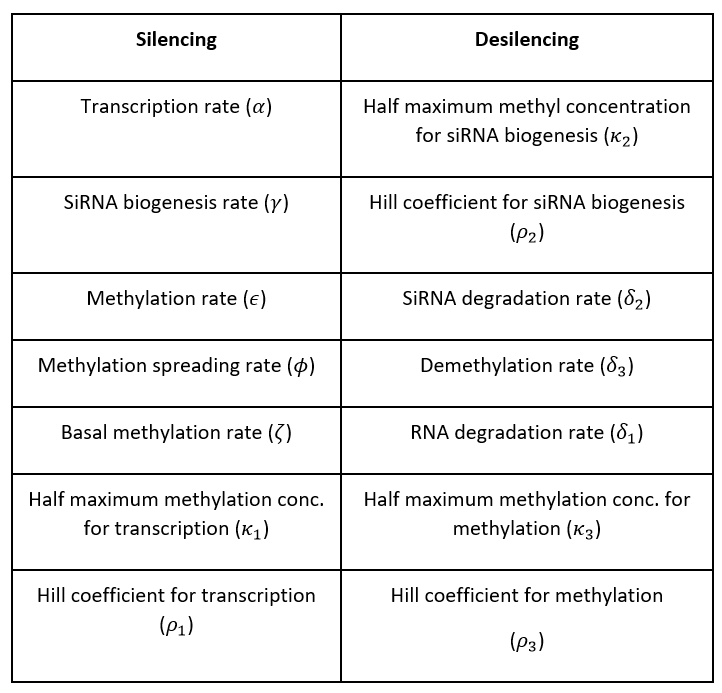

Supplement: S1 Table — (TIF) [file pcbi.1012027.s010.tif]

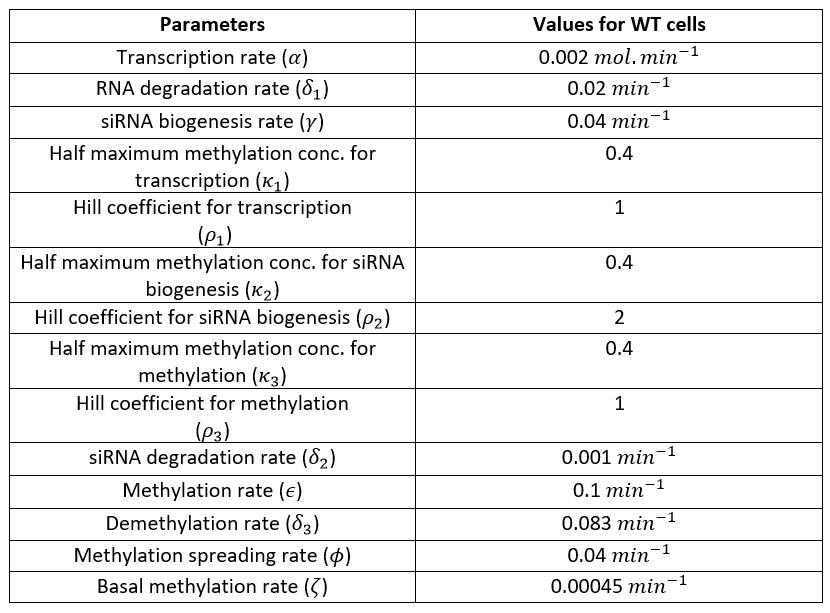

Supplement: S2 Table — (TIF) [file pcbi.1012027.s011.tif]

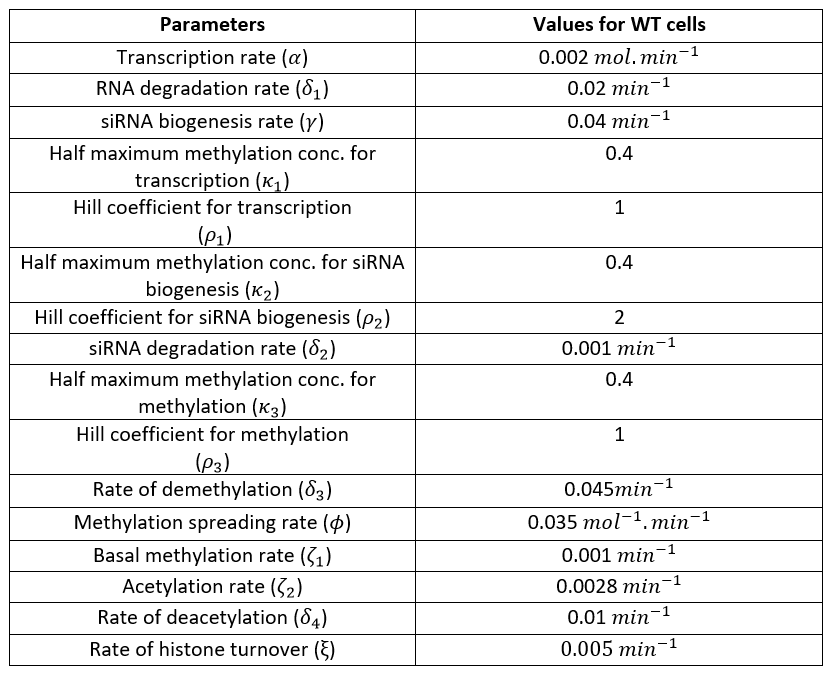

Supplement: S4 Table — (TIF) [file pcbi.1012027.s013.tif]
