# Supplementary material for: Mathematical model for the role of multiple pericentromeric repeats on heterochromatin assembly
Source: PLoS Comput Biol. 2024 Apr 10;20(4):e1012027. doi: 10.1371/journal.pcbi.1012027 (PMC11034663; doi:10.1371/journal.pcbi.1012027)
Supplement: Attachment — Submitted filename: rebuttal.pdf [file pcbi.1012027.s016.pdf]

Reviewer #1: The paper is the first modeling paper on heterochromatin formation where siRNA is the prime factor in nucleosome-mediated epigenetics. The introduction is well written and correctly recapitulates that siRNA is an aspect of obtaining heterochromatin in the pombe mating system in yeast. The central equations present reasonable simple guesses, although they ignore any spatial constraints of reactions within the studied region. The discussion is quite weak, without any robust lessons for the reader.

We thank the reviewer and expanded the discussion to link how the repeat copy number affects pericentromere and rDNA and how the copy number based silencing can act as a sequence-independent defense mechanism.

The paper suggests a model for bistable chromatin where the cooperativity comes from methylated H3K9 directing conversions of RNA to siRNA. Thus it is assumed that double as much methylated nucleosomes increase siRNA production by a factor of 4. How this should work is unclear. Presumably, two random nucleosomes that a methylated have to act in concert to convert an RNA to an siRNA?

In our model, we do assume that siRNA biogenesis rate is dependent on H3K9me in a nonlinear fashion, which is not a simple square of H3K9me fold change. In *S. pombe*, one of key processes in the siRNA biogenesis is the recognition of nascent RNA by the siRNA-bound RITS complex. This leads to the biogenesis of dsRNA and dicer (Dcr1) activities. The RITS complex is made up of Ago1 (argonaute), Chp1 (chromodomain protein) and Tas3. Chp1 is a chromodomain protein, which can bind to H3K9me, and several experimental studies showed that this binding is required for siRNA biogenesis and heterochromatin assembly. The clear quantitation of siRNA biogenesis rate has not been done, and it is difficult to this relationship *in vivo* due to complex network of interaction heterochromatin factors. Additional explanation was added in the page 3 with new references.

Verdel A, Jia S, Gerber S, Sugiyama T, Gygi S, Grewal SI, et al. RNAi-mediated targeting of heterochromatin by the RITS complex. Science. 2004;303(5658):672–6.

Zocco M, Marasovic M, Pisacane P, Bilokapic S, Halic M. The Chp1 chromodomain binds the H3K9me tail and the nucleosome core to assemble heterochromatin. Cell Discov. 2016 Apr 19;2(1):1–15.

Schalch T, Job G, Noffsinger VJ, Shanker S, Kuscu C, Joshua-Tor L, et al. High-affinity binding of Chp1 chromodomain to K9 methylated histone H3 is required to establish centromeric heterochromatin. Mol Cell. 2009 Apr;34(1):36–46.

All consequences of this are correctly analyzed, but overall not surprising. Thus any prediction is a direct consequence of the assumed input, and nothing in the paper demonstrates anything (page 7 " Our study demonstrates that such copy number variation among transcriptionally repressed repeats can alter gene silencing and genomic stability/instability" is an overstatement)

We thank the reviewer for this comment. It is difficult to modify the pericentromeric repeat copy number experimentally, and this is why we rely on a computational model to see how the copy number affects the gene silencing. Our analysis brings a new insight with the emphasis of repeat copy number for heterochromatin formation, which was not tested in fission yeast. On the other hand, our analysis of copy number indicates that unique part (single copy) of the genome will be protected against this type of silencing. In addition, we showed how cell division can

destabilize silencing in Fig 6 and S7, and cells may have evolved to require multiple repeats to stably transmit the silenced pericentromere through cell division. We have toned down the sentence in the main text as “Our quantitative model shows that such copy number variation among transcriptionally repressed repeats can alter gene silencing and chromatin state” (page 7).

Fig. 1 is misleading, there is a catalyzing arrow from unmethylated nucleosomes to RNA to siRNA conversion?

We thank the reviewer for pointing this out, and we modified Fig 1 in a few ways to describe associated processes better. 1) The catalyzing arrow is now from a methylated nucleosome. 2) Spreading of methylation is from a methylated nucleosomes into an unmethylated nucleosomes. 3) We moved the demethylation within the figure.

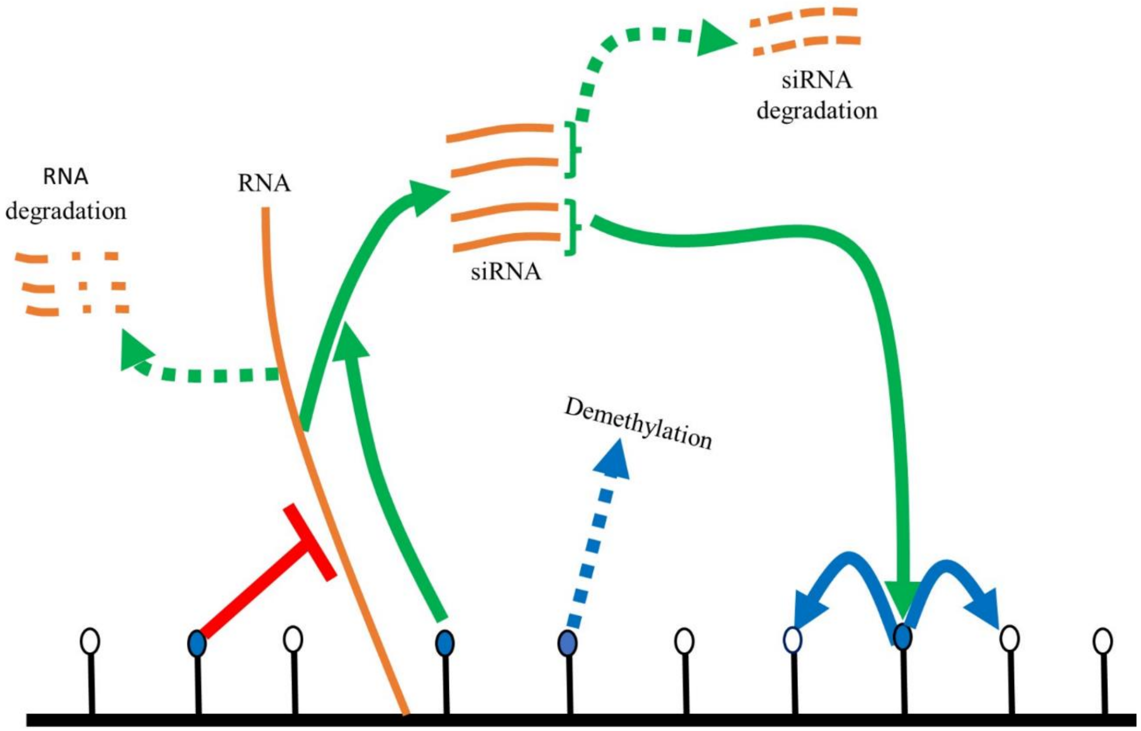

The lateral spreading of H3K9me in Fig. 1 is rather poorly represented by the x3*(1-x3) term in eq. 3, as lateral spreading would change weakly with methylated fraction.

We modified Fig 1 to represent that spreading is between methylated (blue) and unmethylated nucleosomes (white). The spreading of methylation is actually weak compared to the primary targeting mechanism by siRNA and RITS (Motamedi et al (2004) Cell). The deletion of Swi6 and Chp2, which are chromodomain proteins, leads to partial loss of silencing in pericentromere (Motamedi et al (2008) Mol Cell). We modified the main text (Page 3) to include these information. Also, our prediction of the loss of spreading ($\phi = 0$ case in Fig. 5) suggests that some silencing can be maintained.

Motamedi MR, Hong EJE, Li X, Gerber S, Denison C, Gygi S, et al. HP1 proteins form distinct complexes and mediate heterochromatic gene silencing by nonoverlapping mechanisms. Mol Cell. 2008;32(6):778–90.

Motamedi MR, Verdel A, Colmenares SU, Gerber SA, Gygi SP, Moazed D. Two RNAi complexes, RITS and RDRC, physically interact and localize to noncoding centromeric RNAs. Cell. 2004;119(6):789–802.

Motamedi MR, Hong EJE, Li X, Gerber S, Denison C, Gygi S, et al. HP1 proteins form distinct complexes and mediate heterochromatic gene silencing by nonoverlapping mechanisms. Mol Cell. 2008;32(6):778–90.

There is no list of used parameters in the main paper, leaving the reader at a loss what a mere 25% variation in the robustness section was actually accomplished. Importantly if all hill coefficients were equal to 1, then I predict that there would be no bi-stability.

We thank the reviewer for pointing this out. Parameters are included as Table S2, and we pointed it out again in the main text (Page 3).

Table S2. Parameters used for solving differential eqs. (1-3) for WT cell

Parameters	Values for WT cells
Transcription rate constant (α)	0.002 $mol.min^{-1}$
RNA degradation rate constant (δ_1)	0.02 min^{-1}
siRNA biogenesis rate (γ)	0.04 min^{-1}
Half maximum methylation conc. for transcription (κ_1)	0.4
Hill coefficient for transcription (ρ_1)	1
Half maximum methylation conc. for siRNA biogenesis (κ_2)	0.4
Hill coefficient for siRNA biogenesis (ρ_2)	2
Half maximum methylation conc. for methylation (κ_3)	0.4
Hill coefficient for methylation (ρ_3)	1
siRNA degradation rate constant (δ_2)	0.001 min^{-1}
Methylation rate constant (ϵ)	0.1 min^{-1}
Demethylation rate constant (δ_3)	0.083 min^{-1}
Methylation spreading rate constant (ϕ)	0.04 min^{-1}
Basal methylation rate constant (ζ)	0.00045 min^{-1}

We also show that bistability is possible even if all Hill coefficients are 1 (left panel). Intersections of these two curves are steady states (2 stable and 1 unstable). In addition, it is also possible that bistability occurs with $\rho_1 = 0$, $\rho_2 = \rho_3 = 1$ (right panel). These examples show that bistability can occur with a various combination of parameters, and weak cooperativity among interactions can lead to bistability in our model.

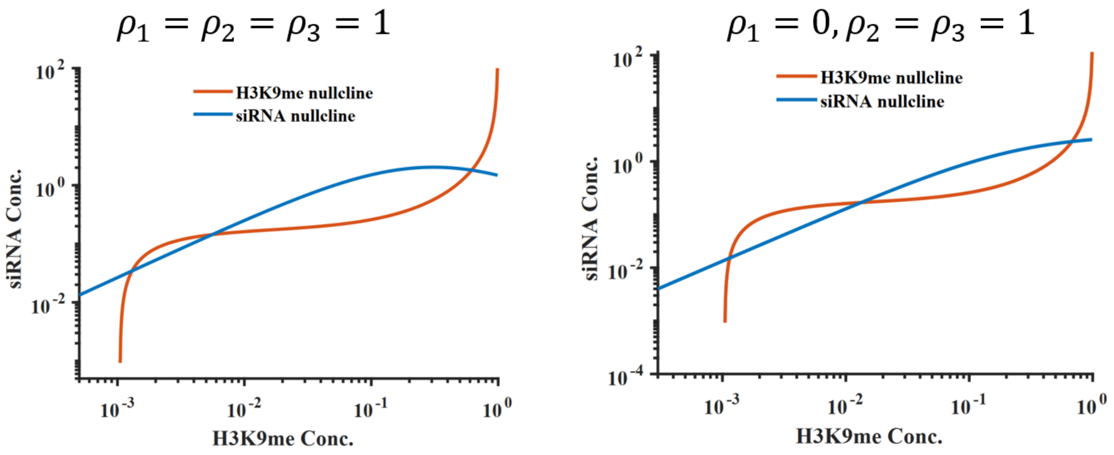

What is the value of the lifetimes of siRNA and RNA respectively, and is there any justification for this?

We used the lifetime of RNA as 50min (the degradation rate = 0.02/min from Table S2) in the absence of siRNA biogenesis. One recent experimental data in yeast suggests that the median RNA lifetime would be 32min (Geisberg et al (2014) Cell), and data from mammalian cells shows that cis-antisense ncRNA (like dg/dh in this study) are more stable than other types of lncRNAs (Clark et al (2012) Genome Res). The lifetime of siRNA is not well known, which we assume to be much more stable than lncRNA (we use 1,000min). This is because most siRNAs are bound by the Argonaute (Ago1), which can inhibit the accessibility of degradation machineries as shown in *Drosophila* (Kingston and Bartel (2021) RNA). We performed RNA- and siRNA-seq in WT cells, and RNA molecules from pericentromeric repeats are found in the siRNA form abundantly with very low lncRNA expression (Joh et al (2016) Mol Cell), suggesting that a low steady level of lncRNA can seed a log of siRNAs.

We included these information in the main text (Page 3).

Geisberg JV, Moqtaderi Z, Fan X, Ozsolak F, Struhl K. Global analysis of mRNA isoform half-lives reveals stabilizing and destabilizing elements in yeast. Cell. 2014 Feb 13;156(4):812–24.

Clark MB, Johnston RL, Inostroza-Ponta M, Fox AH, Fortini E, Moscato P, et al. Genome-wide analysis of long noncoding RNA stability. Genome Res. 2012 May;22(5):885–98.

Kingston ER, Bartel DP. Ago2 protects Drosophila siRNAs and microRNAs from target-directed degradation, even in the absence of 2'-O-methylation. RNA N Y N. 2021 Jun;27(6):710–24.

Joh RI, Khanduja JS, Calvo IA, Mistry M, Palmieri CMCM, Savol AJAJ, et al. Survival in quiescence requires the euchromatic deployment of Clr4/SUV39H by Argonaute-associated small RNAs. Mol Cell. 2016;64(6):1088–101.

The fact that H3K9me represses RNA formation may easily prevent RNA and thus siRNA from being present in heterochromatic state. This must put constraints on some parameters, associated with the transcriptional activity of RNA and the lifetimes of RNA (in particular the value of kappa1 must be large relative to x3?)

We agree with the reviewer that methylation can prevent RNA and siRNA biogenesis. Indeed several previous papers have revealed that there is a steady state where optimal RNA transcription, siRNA generation and H3K9me is required for heterochromatin formation.

Specifically, pericentromere are primarily found in two states: 1) low RNA, high siRNA, and high H3K9me or 2) high RNA, low siRNA, and low H3K9me. This, we think, is in part due to the stability of siRNA as a small amount of RNA can lead the sufficient level of stable siRNAs to maintain heterochromatin. As the reviewer points out, the modulation of kappa1 will increase the RNA, thus siRNA as well, but we can get the natural behavior (low RNA and high siRNA) even if we use kappa1=kappa2=kappa3.

We already discussed this in the main text "Clr4-mediated H3K9 methylation amplify the H3K9me and siRNA signals at heterochromatin. H3K9me, in turn, represses the transcription (30), but even a small number of RNA can be efficiently turned into siRNA" (at the end of siRNA and H3K9me amplification loops on Page 3).

Overall, it is well-motivated to include siRNA as a contribution to chromatin silencing in s. pombe, but the current study is misleading in arbitrarily making this particular reaction the main player. The paper contains too little insights/ideas to justify publication in Plos Computation.

Reviewer #2: This manuscript introduced a mathematical model describing the gene silencing of pericentromeric repeats in which the amount of siRNAs and the level of histone methylation are included as variables, and the copy number of pericentromeric repeats is taken as a parameter. The results show the bistable nature between silenced and desilenced states, and the importance of the copy number on the control of the state. The authors performed the exhaustive bifurcation analysis on the proposed model, and almost no ambiguity is left.

We thank the reviewer for their review and are glad that the reviewer found our paper thorough and clear.

Reviewer #3: In this manuscript the authors describe a mathematical model of histone methylation, focusing on pericentromeric repeats. The authors describe the conditions under which these regions can be stably methylated using a combination of deterministic and stochastic modeling. Overall I found the manuscript well-written, the modeling clearly described, with interesting and thorough analyses, and appropriate conclusions. I have a few minor comments.

I was unclear on whether the pericentromeric repeats are variable across individuals, or within the chromosome itself? The abstract/summary/intro did not specify the type of variability in these repeats. If the variability is within chromosome or across replication events, I'd like to see some discussion by the authors on how this might influence their modeling results, or if the mechanisms of RNAi and siRNA targeting are sensitive to such variability.

We thank the reviewer for pointing this out, and we explained this in the Repeat copy number section (Page 3): "At least one repeat is present on each side of three S. pombe centromeres. The number of repeats varies across chromosomes as chromosome 3 has multiple copies on both side of centromere (32). Each copy can have its own transcriptional activity independent of one another. However, Ago1-bound siRNA can target nascent transcripts across chromosomes and exogenous loci (33), and repeats show similar profiles in gene silencing and H3K9 methylation across chromosomes."

In addition, we expanded the discussions to include how the change in dg/dh and rDNA affect gene silencing and genomic instability.

I did not see the code provided as a supplemental, I would strongly suggest providing the code, at least for the ODE and Gillespie simulations. I would also like to see the version of Matlab used stated in text in the Materials and Methods section.

We now included the Matlab files as a zip file and the version that we used. We included the following sentence at the end of methods "We have used Matlab R2019b and the model codes are included as a supporting file". The supporting file includes the ODE model and Gillespie simulations.

The Gillespie simulation showed the impact of cell division time and noise - is this something that is observed or implicated in disease states? If so, I'd like to see this mentioned in the discussion.

There is not many studies on the effect of noise and how it can be modulated. However, a number of experiments in fission yeast suggest that the facultative heterochromatin can expand in suboptimal growth conditions. H3K9me2 can spread into euchromatic loci when cells enter quiescence (no division) or grow on low temperature (slow division) (Joh et al (2016) Mol Cell; Gallagher et al (2018) Nat Struct Mol Biol). We have included this at the end of stochastic simulation section.

Joh RI, Khanduja JS, Calvo IA, Mistry M, Palmieri CMCM, Savol AJAJ, et al. Survival in quiescence requires the euchromatic deployment of Clr4/SUV39H by Argonaute-associated small RNAs. Mol Cell. 2016;64(6):1088–101.

Gallagher PS, Larkin M, Thillainadesan G, Dhakshnamoorthy J, Balachandran V, Xiao H, et al. Iron homeostasis regulates facultative heterochromatin assembly in adaptive genome control. Nat Struct Mol Biol. 2018 May;25(5):372–83.

I found the title a bit confusing, from what I can gather it seems that methylation influences assembly, but the article doesn't seem to address assembly per se? Perhaps I may be misunderstanding this.

We thank the reviewer for pointing this out. The proper heterochromatin assembly requires the methylation primarily via the RNAi machinery. Therefore, we believe that our model sets up the known state of heterochromatin (low transcription, high siRNA, and high H3K9me in WT case), all these states are affected by the copy number of pericentromeric repeats, and the combination of such states allows the heterochromatin assembly in a copy number dependent manner.
