# Supplementary material for: Mathematical model for the role of multiple pericentromeric repeats on heterochromatin assembly
Source: PLoS Comput Biol. 2024 Apr 10;20(4):e1012027. doi: 10.1371/journal.pcbi.1012027 (PMC11034663; doi:10.1371/journal.pcbi.1012027)
Supplement: Attachment — Submitted filename: rebuttal_2_v2.pdf [file pcbi.1012027.s017.pdf]

Reviewer #1: The revised paper (PCOMPBIOL-D-23-01233R1) meets the minor comments improvement but does not alleviate the main concern:
The model is arbitrary and without any other support that siRNA is a player in H3K9 methylation.

The siRNA biogenesis and RNAi are different in fission yeast from those in higher eukaryotes. The fission yeast lacks H3K27me and DNA methylation, which leaves the H3K9me1/2/3 by a single enzyme Clr4 as the marker for heterochromatin. The argonaute-associated protein complex for RNAi is found in the nucleus, and its function is largely restricted within heterochromatin characterized by H3K9me. RITS (RNA-induced initiation of transcriptional silencing) complex is made up of three proteins, Ago1 (argonaute), Chp1 (HP1 protein with chromodomain for H3K9me), and Tas3, and the RITS complex (specifically Ago1) can bind to siRNAs. Unlike humans where Ago2-mediated RNAi acts in the cytoplasm, there is little evidence that RNAi works in cytoplasm in fission yeast. Disruption of any core proteins of the RNAi pathways (including RITS components, dicer, and RNA-dependent RNA polymerase) leads to the loss of H3K9 methylation and gene silencing within pericentromere and other heterochromatin regions, while euchromatin is largely unaffected by it. Therefore, siRNA and RNAi is widely thought to be the canonical pathway of H3K9 methylation and heterochromatin assembly in fission yeast (Holoch and Moazed (2015) and Allshire et al (2015)). We emphasize this in the main text as "The nucleation of pericentromeric heterochromatin and its spreading by various chromatin-dependent mechanisms is extensively studied in fission yeast where the RNA interference (RNAi) pathway is predominantly responsible for pericentromeric heterochromatin assembly (11–14)." in the 2nd paragraph of introduction (page 2). Fission yeast is one of the most well characterized organisms in terms of heterochromatin assembly, and the RNAi machinery is not as redundant as higher eukaryotes.

The model only considers feedback between histone methylation and siRNA production and assumes that this involves some hill coefficients that are larger than 1. In the answer to previous concerns, the author uses a bunch of references to prove that siRNA plays a role. This is true, and not disputed by anyone. However, there is no evidence of any nonlinearity remotely similar to what is used in the present paper.

Thanks for pointing this out. We are not trying to claim that the nonlinear functions that we use are exact rates, but these match the experimental evidence qualitatively with a small number of parameters as simplifications. Earlier we showed that bistability can emerge with low Hill coefficients (one or two), where reactions are weakly cooperative (some are built-in). In addition, we try to minimize the number of model parameters which would make it easier to generate more complicated dynamical patterns.

$$\dot{x}_1 = \frac{C\alpha}{1+\left(\frac{x_3}{\kappa_1}\right)^{\rho_1}} - \delta_1 x_1 - \frac{\gamma x_1 \left(\frac{x_3}{\kappa_2}\right)^{\rho_2}}{1+\left(\frac{x_3}{\kappa_2}\right)^{\rho_2}} \qquad (1)$$

$$\dot{x}_2 = \frac{\gamma x_1 \left(\frac{x_3}{\kappa_2}\right)^{\rho_2}}{1+\left(\frac{x_3}{\kappa_2}\right)^{\rho_2}} - \delta_2 x_2 \qquad (2)$$

$$\dot{x}_3 = \frac{\epsilon x_2 (1-x_3) \left(\frac{x_3}{\kappa_3}\right)^{\rho_3}}{1+\left(\frac{x_3}{\kappa_3}\right)^{\rho_3}} - \delta_3 x_3 + \phi(1-x_3)x_3 + \zeta(1-x_3) \qquad (3)$$

Hill function (in this case for methylation) generally represents that there are two different rates, at low and high methylation, with some intermediate values in between. The Hill coefficient represents how rapidly the rate changes between the high and low regions. The qualitative behavior of Hill functions is documented with experimental pieces of evidence.

1. 1st term of Eq1: The transcription rate is high, and it decreases when methylation is high. H3K9 methylation is associated with transcriptional silencing in fission yeast.
2. 3rd term of Eq1 (also 1st term of Eq2): The rate of siRNA biogenesis is zero in the absence of RNA, and it becomes saturated with high methylation. The deletion of Clr4 (the only histone methyltransferase for H3K9me1/2/3) leads to the depletion of siRNAs (Gerace et al (2011) and Zhang et al (2011)), which means the siRNA biogenesis rate is high with methylation.
3. 1st term of Eq3: The rate of methylation by siRNA is zero in the absence of methylation, and it becomes saturated with high methylation. The deletion of Chp1 (with the chromodomain for H3K9me binding) largely eliminates the siRNA-mediated methylation, which suggests its recognition of H3K9me is required (Zocco et al (2016) and Schalach et al (2009)).

Again we would like to stress that these nonlinear functions qualitatively match the experimental evidence, and these are simplifications to potentially more complex biological behaviors with a minimal number of model parameters.

The authors further claim that their model gives bistability with all hill coefficients equal to 1 and refer to a figure in the supplement that does not support that. Anyway, this should theoretically be impossible. (as also seen in Fig 4 that considers variations in hill coefficients emphasizing that all lowering of hill coefficients eliminate bistability). Basically, a model without any cooperativity cannot give bistability, and if they can disprove that they would really deserve publication.

Thanks for pointing this out. You are correct that bistability does not occur if all reactions are not cooperative, and the Hill coefficient can be one when the rate of methylation is not cooperative.

However, there are two cooperative interactions built in the model even when the Hill coefficient is one: 1) methylation of unmethylated histones and 2) spreading of methylation.

1. 1st term of Eq3: Although the rate of siRNA-mediated methylation rate is not cooperative, it acts on the unmethylated histones (denoted by $1 - x_3$). Mathematically this term as a whole acts cooperatively, depending on the squared value of methylation (x_3^2). However, this does not lead to bistability.

2. 3rd term of Eq3: The spreading term is proportional to the product of methylated and unmethylated histones. Mathematically this is represented by the squared value of methylation (x_3^2), thus this is cooperative. This term means the entire system has cooperativity built-in, and the steady states (even assuming x_2 is a constant) are described by a 3rd-order polynomial when $\rho_3 = 1$. This is achieved by setting $\frac{\mathrm{d}x_3}{dt} = 0$, which is 2nd order polynomial in the numerator and 1st order polynomial in the denominator. Multiplying both numerator and denominator by the denominator leads to 3rd order polynomial. The resulting 3rd order polynomial can have up to three roots among positive numbers, two of which can be stable steady states, thus bistable.

Therefore, this dynamical system still has some cooperative terms even when all Hill coefficients are one, and it is possible to exhibit bistability.

It is also of some concern that the whole effect of multiple pleirometric repeats comes in because the number of RNA produced in eq. 1 enters in equation 2. What is the justification/mechanism for this particular assumption? This is the only coupling between the repeats, and perhaps the only part of the model that could be reasonably well argued.

Thanks for pointing this out. The copy number of repeats only comes in the form of the overall transcription rate, and this can indirectly feed into the siRNA equation. RNAs can be degraded via two different mechanisms: 1) conventional degradation (2nd term of Eq1; exosome, which we do not track further) and 2) siRNA biogenesis (3rd term of Eq1; through dicer and argonaute). Both terms are linearly proportional to the amount of RNA, which means RNA can go through those two processes with set rates. The rate of conventional degradation is fixed (constant) whereas the rate of conversion into siRNA is methylation-dependent. The chromodomain of Chp1 (part of Ago1-associated RITS complex) is required for the siRNA biogenesis, suggesting the biogenesis is methylation-dependent (Schalach et al (2009)). We have this information in the methods, but also added this in the main text: "RNA can be degraded by or turned into siRNAs in a methylation-dependent manner".

Finally, then why is there exactly zero siRNA produced if there is zero methylated histones, even if there then is plenty of RNA produced (no leaking term)?

Thanks for pointing this out. Our prior study and other papers suggest that the level of siRNA is very low for most coding genes from euchromatins. Considering the high abundance of mRNAs throughout the euchromatin over that of lncRNAs in heterochromatin, a very small leakage can lead to a considerable amount of siRNA from coding genes, which was not observed experimentally. Our previous experiments showed that the fraction of siRNAs from coding genes is less than 1% while the fraction of siRNAs from heterochromatin (pericentromere and telomere combined) is about 80% (Joh et al (2016)). This suggests that the leaky conversion of RNA into siRNA in the absence of methylation is highly unlikely.

In addition, several papers showed that siRNA is almost completely depleted with the deletion of Clr4 (the only histone methyltransferases for H3K9me1/me2/me3) (Gerace et al (2011) and Zhang et al (2011)). This suggests that the conversion of RNA into siRNA requires the H3K9 methylation. We have added the following sentence when describing the siRNA biogenesis: "The siRNA biogenesis requires H3K9me, and siRNAs are depleted with the deletion of Clr4 (15,26)."

Overall, although the paper analyzes the equations reasonably well, there is no reason why these arbitrary equations give new insights into the proposed problem. I do not recommend publication.

Holoch D, Moazed D. RNA-mediated epigenetic regulation of gene expression. Nat Rev Genet. 2015;16(2):71–84.

Allshire RC, Ekwall K. Epigenetic regulation of chromatin states in Schizosaccharomyces pombe. Cold Spring Harb Perspect Biol. 2015;7(7):a018770.

Zocco M, Marasovic M, Pisacane P, Bilokapic S, Halic M. The Chp1 chromodomain binds the H3K9me tail and the nucleosome core to assemble heterochromatin. Cell Discov. 2016 Apr 19;2(1):1–15.

Schalch T, Job G, Noffsinger VJ, Shanker S, Kuscu C, Joshua-Tor L, et al. High-affinity binding of Chp1 chromodomain to K9 methylated histone H3 is required to establish centromeric heterochromatin. Mol Cell. 2009 Apr;34(1):36–46.

Joh RI, Khanduja JS, Calvo IA, Mistry M, Palmieri CM, Savol AJ, et al. Survival in Quiescence Requires the Euchromatic Deployment of Clr4/SUV39H by Argonaute-Associated Small RNAs. Mol Cell. 2016 Dec 15;64(6):1088–101.

Gerace EL, Halic M, Moazed D. The methyltransferase activity of Clr4Suv39h triggers RNAi independently of histone H3K9 methylation. Mol Cell. 2010;39(3):360–72.

Zhang K, Fischer T, Porter RL, Dhakshnamoorthy J, Zofall M, Zhou M, et al. Clr4/Suv39 and RNA quality control factors cooperate to trigger RNAi and suppress antisense RNA. Science. 2011 Mar;331(6024):1624–7.
