# Supplementary material for: Mathematical model for the role of multiple pericentromeric repeats on heterochromatin assembly
Source: PLoS Comput Biol. 2024 Apr 10;20(4):e1012027. doi: 10.1371/journal.pcbi.1012027 (PMC11034663; doi:10.1371/journal.pcbi.1012027)
Supplement: Attachment — Submitted filename: response_round3_v4.docx [file pcbi.1012027.s018.docx]

Reviewer #1: The revised paper ( PCOMPBIOL-D-23-01233R2) does little to alliviate my main concern, that the model is quite arbitrary, and only justified by papers that prove the importance of methylation via siRNA. However also deacetylation H3K9 is crucial, acting through the deacetylases Clr3 or Sir2 (see below list of references). This two-step process has already been included in standard models in the epigenetics field for the last 15 years (some references below).

We would like to thank you for bringing this important role of HDAC and acetylation. We are not downplaying the critical role of HDACs, and we have shown that the HDAC activity is the prerequisite of methylation (Khanduja et al in press Cell). In the main model, we tried to present testable variable as RNA, siRNA, and H3K9 methylation (me1/me2/me3) are often probed in *S. pombe* studies. H3K9ac (possible) and unmodified H3K9 (limited antibody) can be quantified experimentally in a specific assays, but prediction on these may not be difficult to test experimentally.

We can certainly include this as a part of our quantitative model, but we have to be careful with modeling HDAC-mediated methylation. In fact, HDACs like Sir2 and Clr3 are clearly associated with silencing in pericentromere, which we have shown by the loss of silencing upon deletion experimentally (Khanduja et al in press Cell). However, the experimental evidence for HDAC activity of Clr3 on H3K9ac is still lacking, and several papers suggest that the substrate of Clr3 is H3K14ac, and the HDAC enzymatic activity is crucial for H3K9me (Yamada et al 2005 and Watts et al 2018).

To incorporate this alternative model, we added a section with a) a description of alternative model with acetylated, unmodified, and methylated histones (H3K9). In addition, we added two new supplementary figures (Figures S8-9) to explain these results. In summary, the added 5-state models (RNA/siRNA/acetylated histone/unmodified histone/methylated histone) can recapitulate the behaviors of our main model, in terms of copy-number-dependent silencing and bistability (Figure S8-9). When the HDAC activity is reduced (deletion of HDACs), the desilenced state is favored regardless of the copy number.

We assume that new histones are acetylated, which needs to be deacetylated prior to potential methylation (Alabert et al (2015) Genes Dev). We assumed a set rate for acetylation, deacetylation, and histone turnover. This alternative model does not change the model’s behavior significantly (Figure S9AIn addition, this model suggests that acetylation (combined effect of all HDACs) is required for methylation (Figures S9B-C). Figure S9B illustrates the partial loss of HDAC activity, which was reported to be bistable (Alter et al 2012). Figure S9C is in agreement with our experimental data that H3K9me in heterochromatin is almost completely lost in the double deletion of *sir2* and *clr3* (Khanduja et al in press Cell).

References:

JS Khanduja, RI Joh, JA Paulo, CM Palmieri, J Zhang, AOD Gulka, W Hass, SP Gygi, and M Motamedi. RNA quality control factors nucleate Clr4/SUV39H and trigger constitutive heterochromatin assembly (in press *Cell*)

BJ Alper , BR Lowe, JF Partridge. Centromeric heterochromatin assembly in fission yeast—balancing transcription, RNA interference and chromatin modification. Chromosome Res. 2012;20(5):521–34.

BR Watts, S Wittmann, M Wery, C Gautier, K Kus, A Birot, DH Heo, C Kilchert, A Morillon, L Vasiljeva. (2018) Histone deacetylation promotes transcriptional silencing at facultative heterochromatin. NAR 46(11): 5426-5440.

C Alabert, TK Barth, N Reveron-Gomez, S Sidoli, A Schmidt, ON Jensen, A Imhof, A Groth. (2015) Two distinct modes for propagation of histone PTMs across the cell cycle. Genes Dev. 15(29): 585-590.

I am a big supporter of simple models, so all in favour of removing details. But in this case I
believe that you have thrown the baby out with the water. Of smaller note, then your argument that eq. 3 gives bistability with constant x2 does not sound true: The spreading term x3*(1-x3) cannot give bistability, as it never provides a steeper than proportional increase of x3 with x3.
However, there might be some room in parameter space if siRNA dynamics are so fast that x2 is proportional to x3 when inserted in the first term of eq. 3.

As you explained, a quadratic function cannot intersect with a straight line more than twice. However, the first term in eq3 is not linear, and it is in the form of x/(kappa+x). A quadratic function can intersect with such a Hill function (with Hill coefficient 1) up to three times. With three possible steady states, two can be stable equilibrium, which results in bistability.

Blue and red curves represent $y=3x(1-x)$ and $y=\frac{x}{0.2+x}$, respectively. These two curves can intersect at three distinct points.

In any case, the whole analysis in the paper uses the Hill coefficient rho2=2 without any justification (How could one possibly imagine a mechanism where double the fraction of methylated nucleosomes could give four times the number of siRNA. Also, no experimental references were given to support that basic assumption). Therefore the paper is too misleading to be recommended for publication.

Our Hill function terms are in the standard Hill equation form, which means rho2=2 does not lead to 4-fold change in the siRNA-biogenesis rate. This would be true without the denominator, but the term $\left( \frac{x_{3}}{\kappa_{2}} \right)^{\rho_{2}}/\left( 1+\left( \frac{x_{3}}{\kappa_{2}} \right)^{\rho_{2}} \right)$ from eq2 is less than 1. In fact, when rho2=2, the rate of siRNA biogenesis can increase by 60% even with doubling the methylation (from $\frac{1^{2}}{1+1^{2}}=\frac{1}{2}=0.5$ to $\frac{2^{2}}{1+2^{2}}=\frac{4}{5}=0.8$). There are no papers that try to measure the Hill coefficient, but there are evidences showing that the rate is nonlinear and saturating at high methylation. Rho2 is the parameter which determines how sharply siRNA biogenesis rate is saturating. In general, high Hill coefficient can promote multi-stability, and we thought rho2=2 would be a realistic value, which is nonlinear and weakly saturating with methylation.

Review of some of the models (some of which specialize in H3K9 in S.Pombe):
O’Kane, Callum J., and Edel M. Hyland. "Yeast epigenetics: the inheritance of histone modification states." Bioscience Reports 39.5 (2019): BSR20182006.
for more details see also:
Dodd, Ian B., et al. "Theoretical analysis of epigenetic cell memory by nucleosome modification." Cell 129.4 (2007): 813-822.
Angel, Andrew, et al. "A Polycomb-based switch underlying quantitative epigenetic memory." Nature 476.7358 (2011): 105-108.
M. Ancona, et al., Competition between local erasure and long-range spreading of a single biochemical mark leads to epigenetic bistability, Phys. Rev. E 101, 042408 (2020).
A. Z. Abdulla et al. Painters in chromatin: a unified quantitative framework to systematically characterize epigenome regulation and memory, Nucleic Acids Res. 50, 9083 (2022).

References showing the requirement for the histone deacetylase Clr3 for heterochromatic gene silencing :

Histone deacetylase homologs regulate epigenetic inheritance of transcriptional silencing and chromosome segregation in fission yeast. Grewal SI, Bonaduce MJ, Klar AJ.Genetics. 1998 Oct;150(2):563-76. doi: 10.1093/genetics/150.2.563.PMID: 9755190
The nucleation and maintenance of heterochromatin by a histone deacetylase in fission yeast. Yamada T, Fischle W, Sugiyama T, Allis CD, Grewal SI.Mol Cell. 2005 Oct 28;20(2):173-85. doi: 10.1016/j.molcel.2005.10.002.PMID: 16246721
Three additional linkage groups repress transcription and meiotic recombination in the mating-type region of Schizosaccharomyces pombe. Thon G, Cohen A, Klar AJ.Genetics. 1994 Sep;138(1):29-38. doi: 10.1093/genetics/138.1.29.PMID: 8001791

Mutations in rik1, clr2, clr3, and clr4 genes asymmetrically derepress the silent mating-type loci in fission yeast. Ekwall K, Ruusala T.Genetics. 1994 Jan;136(1):53-64. doi: 10.1093/genetics/136.1.53.PMID: 8138176)
References showing a requirement for the histone deacetylase Sir2 in heterochromatin:
Sir2 regulates histone H3 lysine 9 methylation and heterochromatin assembly in fission yeast. Shankaranarayana GD, Motamedi MR, Moazed D, Grewal SI.Curr Biol. 2003 Jul 15;13(14):1240-6. doi: 10.1016/s0960-9822(03)00489-5.PMID: 12867036
Distinct roles for Sir2 and RNAi in centromeric heterochromatin nucleation, spreading, and maintenance. Buscaino A, Lejeune E, Audergon P, Hamilton G, Pidoux A, Allshire RC.EMBO J. 2013 May 2;32(9):1250-64. doi: 10.1038/emboj.2013.72. Epub 2013 Apr 9.PMID: 23572080

Sir2 is required for Clr4 to initiate centromeric heterochromatin assembly in fission yeast. Alper BJ, Job G, Yadav RK, Shanker S, Lowe BR, Partridge JF.EMBO J. 2013 Aug 28;32(17):2321-35. doi: 10.1038/emboj.2013.143. Epub 2013 Jun 14.PMID: 23771057
(Comment in: Histone deacetylases govern heterochromatin in every phase. Murakami Y.EMBO J. 2013 Aug 28;32(17):2301-3. doi: 10.1038/emboj.2013.154. Epub 2013 Jul 5.PMID: 23832177)

Thanks for suggesting these papers. We have included them through the main text.

Reviewer #4: In this manuscript, Ghimire et al. present a mathematical model for the relationship between pericentromeric repeat copy number and heterochromatin assembly. Their work introduces an important new variable into a model for silencing in pericentromeric DNA repeats. Previous modeling attempts have relied on histone modification feedback loops, the formation of condensates, and competition between activating and repressing histone modifications. Taking copy number of repeats into account is a valuable new addition that should be of general interest to the field.

I have also gone over the comments of reviewer #1 which raises some important points about the modeling effort. I feel like the authors have provided reasonable answers to the reviewer's concerns. While the model contains some arbitrary parameters, it also makes predictions regarding the importance of copy number that can be experimentally tested in the future in S. pombe and other systems. As such, it is likely to stimulate new thinking that will impact future modeling efforts dealing with epigenetic inheritance and bistability as well as new lines of experimentation.

The mathematical modeling also relies on a deep knowledge of RNAi-mediated heterochromatin formation and is consistent with the well-established genetic requirements for this process in S. pombe. As noted above, the predictions of the model regarding copy number and its relationship to H3K9me and siRNA concentration are testable (although beyond the scope of the present study) and will stimulate future studies.

The authors should modify their title to indicate their study is a modeling effort. For example, “A mathematical model for the role of …. “

We would like to thank you for your comments. We have modified the title of the manuscript as “Mathematical model for the role of multiple pericentromeric repeats on heterochromatin assembly” and added a section for an alternative model to consider the role of HDAC and acetylated/unmodified histones.
